# molBLOCKS: decomposing small molecule sets and uncovering enriched fragments

**DOI:** 10.1093/bioinformatics/btu173

**Published:** 2014-03-28

**Authors:** Dario Ghersi, Mona Singh

**Affiliations:** ^1^Lewis-Sigler Institute for Integrative Genomics and ^2^Department of Computer Science, Princeton University, Princeton, NJ 08544, USA

## Abstract

**Summary:** The chemical structures of biomolecules, whether naturally occurring or synthetic, are composed of functionally important building blocks. Given a set of small molecules—for example, those known to bind a particular protein—computationally decomposing them into chemically meaningful fragments can help elucidate their functional properties, and may be useful for designing novel compounds with similar properties. Here we introduce molBLOCKS, a suite of programs for breaking down sets of small molecules into fragments according to a predefined set of chemical rules, clustering the resulting fragments, and uncovering statistically enriched fragments. Among other applications, our software should be a great aid in large-scale chemical analysis of ligands binding specific targets of interest.

**Availability and implementation:** molBLOCKS is available as GPL C++ source code at http://compbio.cs.princeton.edu/molblocks.

**Contact:** mona@cs.princeton.edu

**Supplementary information:** Supplementary data are available at *Bioinformatics* online.

## 1 INTRODUCTION

Endogenous small molecules are synthesized in the cell in a modular fashion, using building blocks or fragments that are often conserved across organisms (Muto *et al.*, 2007). Fragment-based drug discovery has also emerged as an important paradigm to navigate the diversity of the chemical landscape and to profile protein druggability (Hajduk and Greer, 2007). Further, it has been shown that the toxicity of certain drugs can be explained by the presence in their structure of fragments that are shared by toxic compounds (Ahmed *et al.*, 2011). Although many programs are available to assemble small molecules from fragments (Schneider and Baringhaus, 2013), the reverse problem of breaking down small molecules and analyzing the corresponding fragment sets has been studied less extensively. An implementation of the RECAP algorithm (Lewell *et al.*, 1998) to fragment small molecules can be found in a commercial program (fragmenter, www.chemaxon.com), and is available in the RDKit library (http://www.rdkit.org), which also implements the BRICS fragmentation algorithm (Degen *et al.*, 2008). However, given a diverse set of small molecules that share a property of interest, there is no automated tool to identify statistically enriched fragments that might explain their activity.

**Fig. 1.**
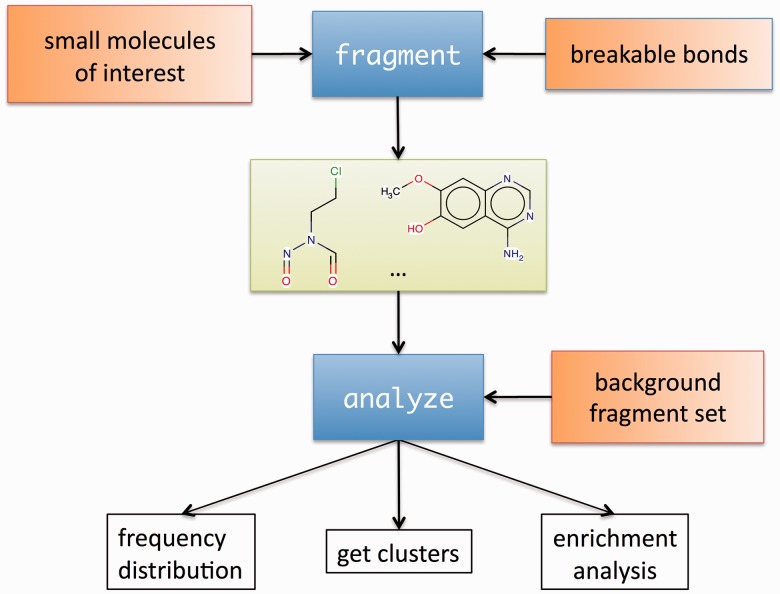
The fragment program takes as input a set of small molecules and user-defined rules that specify the bonds to break, and then applies these rules to fragment the molecules. As an optional second step, carried out by the analyze program, the user can cluster the fragments and/or determine whether the frequency of any of the fragments is enriched as compared with a background set of fragments

Here we introduce the molBLOCKS suite, which allows users to break down small molecules into chemically meaningful fragments and analyze the resulting fragment distribution (Fig. 1). The software consists of two command-line programs: fragment and analyze. The fragment program reads user-defined rules to specify the bonds to break or uses default sets of rules [RECAP (Lewell *et al.*, 1998), CCQ [www.chemaxon.com], and BRICS (Degen *et al.*, 2008)]. Then, the program applies these rules to fragment the molecules, and generates all fragments with a number of heavy atoms above a minimum size defined by the user.

The analyze program collects statistics on the frequency with which each fragment occurs, clusters fragments using a user-defined similarity threshold based on a fingerprint representation (O’Boyle *et al.*, 2011) of the fragments and selects a representative fragment for each cluster. This program can also perform enrichment analysis at the level of either fragments or clusters.

A typical scenario where fragment and enrichment analyses can be applied is when dealing with a library of small molecules, a subset of which has a specific property of interest. In these cases, molBLOCKS can be used to fragment the whole library and determine which (if any) fragments are significantly enriched in the set with the property of interest. Fragmentation and enrichment analysis of small molecules may also be useful in analyzing proteins. For example, ligands bound by proteins that share a common property, such as a specific function, can be analyzed in this manner. Such an approach would provide a complement to the functional enrichment analyses that are routinely performed with Gene Ontology terms (Huang da *et al.*, 2009).

Extensive fragmentation of the entire DrugBank (Wishart *et al.*, 2006) collection of 6460 small molecules with the default rules took 53 s on an iMac with a 2.66 GHz processor. A user’s guide with implementation details and more tests is provided with the suite.

## 2 METHODS

### 2.1 fragment

Small molecules and bond-breaking rules are specified with SMILES (Weininger, 1988) and SMARTS (Daylight Inc.) notation, respectively. The open-source Open Babel C API (O’Boyle *et al.*, 2011) is used to process the SMILES and SMARTS notation. To ensure that all possible fragments of a minimum given size are generated (extensive fragmentation, which can be turned on with the -e flag), the program uses the following strategy. Cleavable bonds are represented as nodes in an undirected graph, with an edge between two nodes if both bonds can be cut; we note that not all bonds that match the rules can be cleaved at the same time, because doing so would yield fragments smaller than the minimum size. Subsequently, the Bron–Kerbosch algorithm (Bron and Kerbosch, 1973) is used to identify all maximal cliques (i.e. all sets of bonds that can be cleaved simultaneously). Finally, all possible fragments are generated by cutting the bonds within each maximal clique, one clique at a time. Without extensive fragmentation, the program returns only one possible set of fragments.

### 2.2 analyze

#### 2.2.1 Fragment frequency

The program returns a frequency distribution with the total number of molecules that contain a given fragment. Multiple instances of the same fragment in a molecule are counted only once.

#### 2.2.2 Fragment clustering

Fragments are first converted to the Open Babel (O’Boyle *et al.*, 2011) default FP2 fingerprint representation, which is based on linear segments of up to seven atoms in length. The Tanimoto coefficient between the fingerprint representations of two fragments is used to compute their fragment similarity. For a given threshold of similarity, a graph is created where there is a node for each fragment, and an edge between two nodes whose corresponding fragments are considered similar. Subsequently, the analyze program extracts the connected components of the graph, and selects a representative element for each cluster as the fragment with the highest average similarity to all the other fragments in the cluster.

#### 2.2.3 Enrichment analysis

Enrichment analysis can be carried out to identify whether specific fragments (or clusters of fragments) appear in a set of molecules more frequently than expected by chance, as compared with a background set of fragments. The hypergeometric distribution was chosen to model the probability of obtaining a number of fragments (or clusters of fragments) equal to or greater than the observed by chance alone, in analogy to what is routinely done in Gene Ontology enrichment analyses (Rivals *et al.*, 2007). The analyze program returns both uncorrected *P*-values and FDRs obtained with the Benjamini–Hochberg procedure (Benjamini and Hochberg, 1995) to handle multiple hypothesis testing.

## 3 USAGE

As an example of how to use the molBLOCKS suite, we fragmented a set of antineoplastic drugs extracted from KEGG (Kanehisa *et al.*, 2012) with the following command:

fragment -i antineoplastic.smi -r RECAP.txt

      -n 4 -o antineoplastic.frag –e

where antineoplastic.smi is a text file containing the small molecules in SMILES format to fragment. The RECAP.txt file contains a definition of the cleavable bonds, encoded as SMARTS patterns. The −e flag specifies extensive fragmentation, and the −n parameter controls the minimum size of a fragment, defined as the total number of heavy atoms. The antineoplastic.frag file contains the output of the fragmentation.

Subsequently, we identified the enriched fragments in a background dataset of drugs in KEGG with the analyze program:

analyze -i antineoplastic.frag -c 0.8

     -e background.frag -o distr.txt

**Fig. 2.**
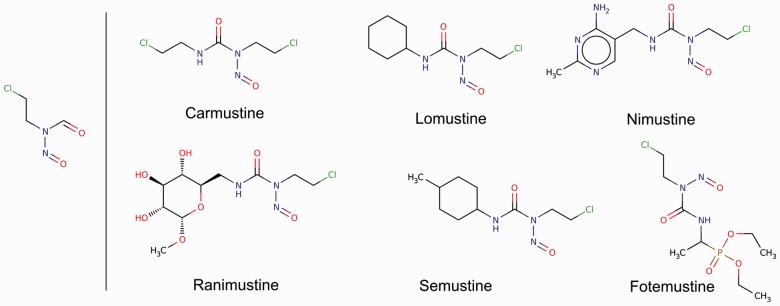
Antineoplastic (i.e. tumor inhibitor) drugs were fragmented and analyzed with molBLOCKS. Four clusters of fragments were found to be enriched in this set of 165 drugs. The representative fragment for the first cluster is shown in the left panel, and drugs that contain a fragment in this cluster are shown in the right panel. These compounds are alkylating agents, which damage DNA by attaching an alkyl group to the guanine base. The enriched fragment comes from nitrosurea, the molecule from which these compounds derive. Molecules are visualized with Marvin Sketch (http://www.chemaxon.com/products/marvin/marvinsketch/). The remaining enriched clusters are given in the Supplementary Materials

With the optional −c parameter, analyze clusters the fragments at the specified Tanimoto coefficient. The optional −e parameter specifies the background set for enrichment analysis; this set must contain the fragments in the input set for the results to be meaningful. Figure 2 shows an example of an enriched fragment and its parent molecules in the antineoplastic set. See the Supplementary Materials for further details.

## Supplementary Material

Supplementary Data
