# Supplementary material for: molBLOCKS: decomposing small molecule sets and uncovering enriched fragments
Source: Bioinformatics. 2014 Mar 28;30(14):2081–3. doi: 10.1093/bioinformatics/btu173 (PMC4080744; doi:10.1093/bioinformatics/btu173)
Supplement: Supplementary Data [file supp_30_14_2081__index.html]

molBLOCKS: decomposing small molecule sets and uncovering enriched fragments — molBLOCKS: decomposing small molecule sets and uncovering enriched fragments — Supplementary Data 

# molBLOCKS: decomposing small molecule sets and uncovering enriched fragments

## Supplementary Data

files

**Files in this Data Supplement:**

- Supplementary Data - pdf file
- Supplementary Data - pdf file
