# Supplementary material for: molBLOCKS: decomposing small molecule sets and uncovering enriched fragments
Source: Bioinformatics. 2014 Mar 28;30(14):2081–3. doi: 10.1093/bioinformatics/btu173 (PMC4080744; doi:10.1093/bioinformatics/btu173)
Supplement: Supplementary Data [file supp_btu173_molBLOCKSGuide.pdf]

molBLOCKS– User's Guide

Dario Ghersi

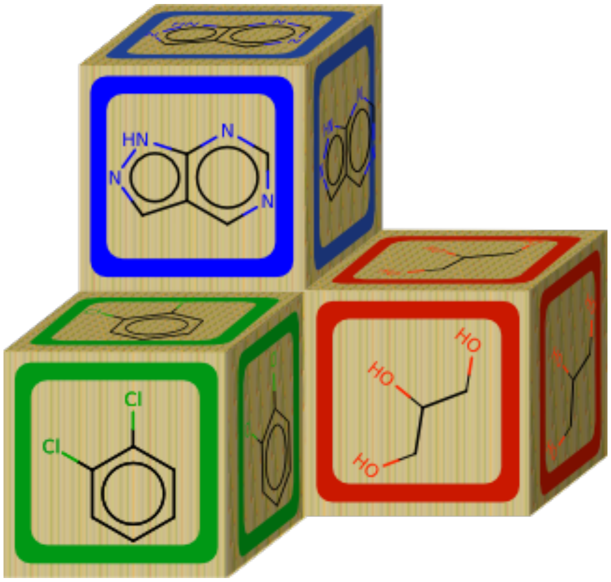

Copyright © 2014 Dario Ghersi

HTTP://COMPBIO.CS.PRINCETON.EDU/MOLBLOCKS

January 2014

Disclaimer and Acknowledgements

These programs are distributed in the hope that they will be useful, but without any warranty; without even the implied warranty of merchantability or fitness for any purpose. The entire risk as to the quality and performance of the program is with the user.

The `molBLOCKS` suite was developed by Dario Ghersi in Mona Singh's lab at the Lewis-Sigler Institute for Integrative Genomics, Princeton University.

Email addresses:
Dario Ghersi: `dario.ghersi@princeton.edu`

Contents

1 Introduction 5

1.1 Overview of `molBLOCKS` 5

1.2 Representing small molecules with the SMILES notation 6

1.2.1 Atoms and bonds 6
1.2.2 Branches and cycles 7
1.2.3 Stereochemistry 7
1.2.4 Canonical form 8

1.3 Defining rules with SMARTS 8

1.3.1 Specifying atoms 9
1.3.2 Specifying bonds 9
1.3.3 Logical operators 9
1.3.4 Examples of SMARTS patterns 9

2 Installing the `molBLOCKS` suite 11

2.1 Compiling `molBLOCKS` in Linux and Mac OS X 11

2.2 Running `molBLOCKS` in a Virtual Machine 12

3 The `fragment` program 13

3.1 Using the `fragment` program 13

3.1.1 Input files 13
3.1.2 Parameters 14
3.1.3 Output 15

3.2 Under the hood 16

4 The `analyze` program 17

4.1 Using `analyze` 17

4.1.1 Input files 17

4.1.2 Parameters 17

4.1.3 Output 18

4.2 A tutorial on fragment clustering and enrichment analysis 19

4.3 Under the hood 20

4.3.1 Fragment clustering 20

4.3.2 Enrichment analysis 21

Bibliography 23

Overview of molBLOCKS
Representing small molecules with the SMILES notation
Atoms and bonds
Branches and cycles
Stereochemistry
Canonical form
Defining rules with SMARTS
Specifying atoms
Specifying bonds
Logical operators
Examples of SMARTS patterns

1 — Introduction

1.1 Overview of molBLOCKS

The molBLOCKS suite allows users to break down small molecules into chemically meaningful fragments and to analyze the resulting fragment distribution (see Figure 1.1). molBLOCKS consists of two programs: `fragment` and `analyze`.

The `fragment` program reads user-defined rules to specify the bonds to break, or uses the default set of rules based on the RECAP algorithm [Lew+98]. Then, the program applies these rules to fragment the molecules, and exhaustively generates all fragments above a minimum size that is defined by the user.

The `analyze` program provides users with the option of analyzing the fragments yielded by `fragment`. Besides collecting statistics on the frequency of each fragment, the `analyze` program also clusters fragments with a user-defined similarity threshold that is based on a fingerprint representation of the fragments. The program then selects the most representative fragment from a cluster as the fragment with the highest average similarity to every other fragment in its cluster. Another feature provided by the `analyze` program is enrichment analysis. Let us suppose we are dealing with a library of small molecules, a subset of which has a specific property of interest. We can then fragment the whole library with the `fragment` program, and determine which (if any) fragments are significantly enriched in the set with the property of interest. The enrichment analysis can also be carried out at the level of clusters.

The following sections will briefly describe the SMILES and SMARTS formats used by molBLOCKS to define the molecules and the bonds to break. More information about SMILES and SMARTS can be found on the DAYLIGHT website. [1] [2]

[1] http://www.daylight.com/dayhtml/doc/theory/theory.smiles.html
[2] http://www.daylight.com/dayhtml/doc/theory/theory.smarts.html

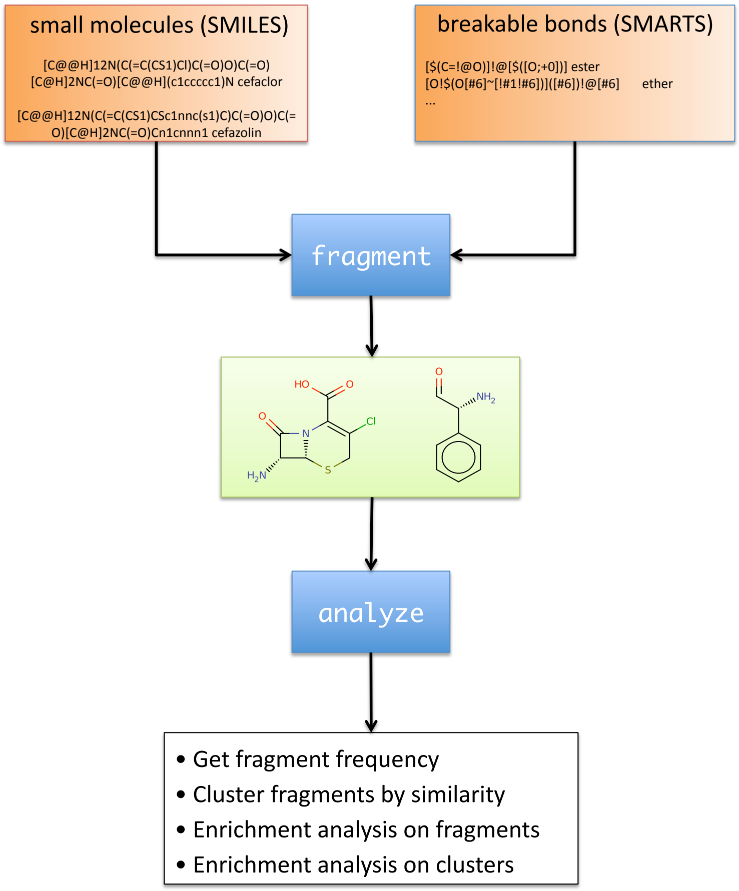

Figure 1.1: **Flowchart for the `molBLOCKS` suite.** The `fragment` program reads user-defined rules that specify the bonds to break, and then applies these rules extensively to fragment the molecules. As an optional second step – carried out with the `analyze` program – the user can perform a variety of analyses on these fragments, such as cluster or enrichment analysis.

1.2 Representing small molecules with the SMILES notation

`molBLOCKS` uses the SMILES (Simplified Molecular Input Line Entry System) [Wei88] notation to represent small molecules. Most chemoinformatics and bioinformatics databases (e.g., DrugBank [Wis+06], and the PDB [Ber+00]) provide SMILES codes for small molecules. The `openbabel` program [3] easily converts small molecules from other formats (e.g., MOL, PDB, and SDF) into SMILES strings.

1.2.1 Atoms and bonds

In SMILES, atoms are specified by their chemical symbols, enclosed in square brackets. For atoms belonging to the organic subset (B, C, N, O, P, S, F, Cl, Br, and I) the square brackets are usually omitted. Atoms in aromatic rings are written lower-case (e.g., an aromatic carbon would be **c**,

[3] http://openbabel.org

whereas an aliphatic carbon would be written as **C**). Hydrogen atoms are implied and need not be explicitly notated, but are required in the presence of square brackets (e.g., **[NH3]**).

Single bonds are represented by a dash, **-**, but are usually omitted. Double and triple bonds are represented by the **=** and **#** symbols, respectively. For example, a molecule like ethanol – which contains no double or triple bonds – could be represented simply as **CCO** (Figure 1.2A), whereas ethylacetylene – which contains one triple bond – can be written as **C#CCC** (Figure 1.2B).

1.2.2 Branches and cycles

Branches are enclosed in parentheses, and connect to the left. For example, isopropyl alcohol can be written as **CC(O)C** (Figure 1.2C). Cyclic structures are specified by adding the same label to atoms that are non-adjacent in the SMILES string, but are connected in the molecule. For example, cyclohexane can be written as **C1CCCCC1** (Figure 1.2D).

1.2.3 Stereochemistry

E and Z isomerism is described with the **/** and **** characters. For example, cys-2-butene would be represented as **C/C=C\C** (Figure 1.2E), whereas trans-2-butene (with the methyl groups on the opposite side of the double bond) would be **C/C=C/C** (Figure 1.2F). The @ and @@ characters are used to specify the chirality of a tetrahedral carbon. The @ and @@ characters indicate that the substituents appear clockwise and anti-clockwise, respectively, when looking from the first neighbor of the chiral atom listed in the SMILES string.

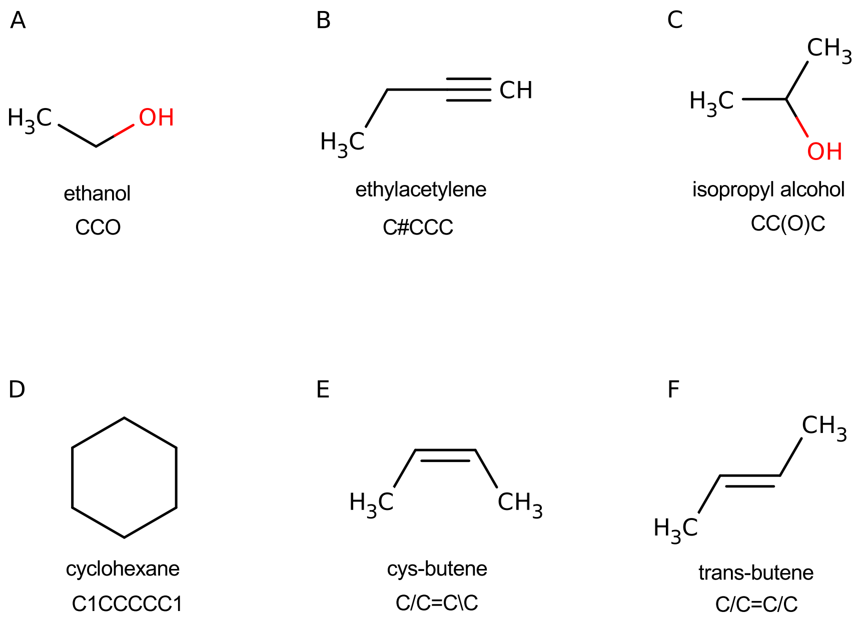

Figure 1.2: **Examples of simple small molecules, represented as SMILES strings.**

1.2.4 Canonical form

In general, more than one SMILES string can correspond to the same small molecule. For example, these are all correct SMILES strings that represent ethanol:

- **CCO**
- **OCC**
- **C(O)C**
- **[CH3][CH2][OH]**

`fragment` accepts any SMILES string, as long as it is correct. The output, however, is in canonical form. In other words, identical fragments will be output as the same SMILES string, even if they had been written differently in the molecules they came from. The canonicalization algorithm is part of the Open Babel library [OBo+11] used by `fragment`. Figure 1.3 shows some examples of small molecules of biological and pharmacological interest, represented as SMILES strings.

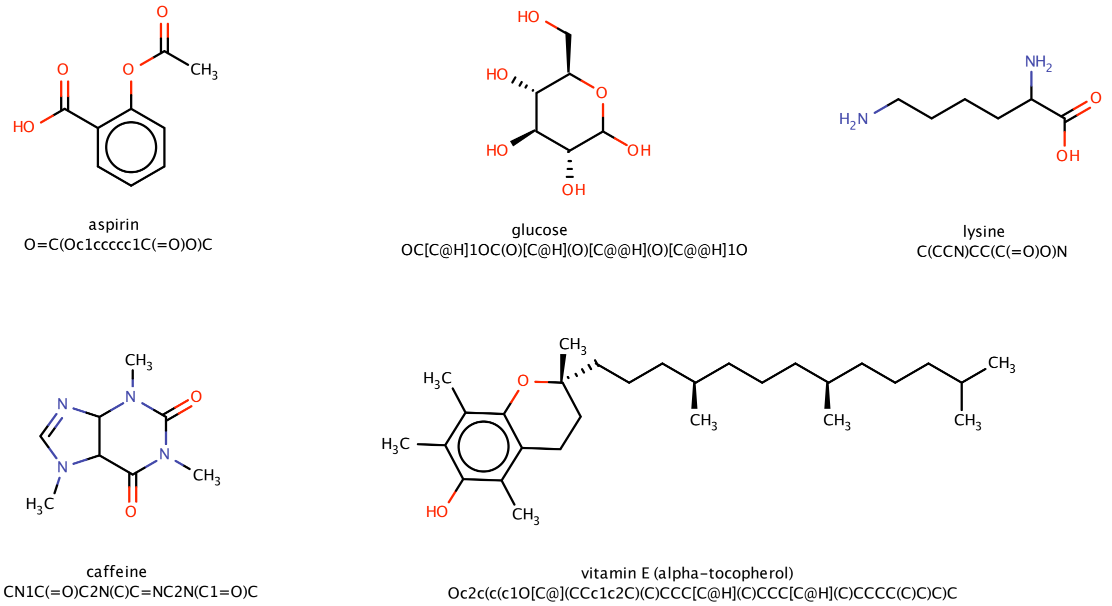

Figure 1.3: **Examples of small molecules of biological and pharmacological interest, represented as SMILES strings.**

1.3 Defining rules with SMARTS

The `fragment` program requires a set of rules that specifies the bonds that can be cleaved. These rules have to be encoded as SMARTS (SMiles ARbitrary Target Specification) patterns that specify the two atoms that define the bond to be cleaved. SMARTS is a format created by `Daylight Chemical Information Systems, Inc.` for matching substructures and properties, and the rules are an extension of the SMILES notation (all SMILES symbols are valid in SMARTS).

1.3.1 Specifying atoms

Atoms can be specified as in SMILES strings, or by their atomic number preceded by the **#** symbol (e.g., **#6** would match *any* carbon, either aliphatic or aromatic), or by their atomic mass, within **<>** . The wildcard * represents any atom, while the **a** and **A** symbols represent aromatic and aliphatic atoms, respectively.

1.3.2 Specifying bonds

Bonds are specified as in SMILES strings, with some additional symbols. The ~ symbol indicates any bond, the **:** symbol represents an aromatic bond, while the @ symbol indicates any ring bond.

1.3.3 Logical operators

SMARTS allows the use of logical operators to combine expressions. The **!** symbol negates an expression, the **&** and **;** symbols represent high-precedence and low-precedence boolean **and** operators, respectively. The boolean **or** operator is represented by a comma (**,**).

1.3.4 Examples of SMARTS patterns

Some examples of SMARTS pattern used to encode the default RECAP rules follow:

[c]!@[c] aromatic carbon – aromatic carbon bond

[#6]=!@[#6] olefin bond

[$(C=!@O)]!@[$([O;+0])] ester bond

Note that the **!@** symbols separate the two atoms that define the bond to cleave, and prevent bond cleavage from occurring in a ring. The last example introduces two new symbols: the **+** symbol, that specifies the formal charge of an atom, and the **$** symbol, which is used to define recursive SMARTS expressions.

Compiling molBLOCKS in Linux and Mac OS X
Running molBLOCKS in a Virtual Machine

2 — Installing the molBLOCKS suite

molBLOCKS has only two external dependencies: the boost library [SLL02] and the openbabel library [OBo+11]. Both are provided in the download package. The boost library is header based, and requires no installation, whereas openbabel needs to be compiled and installed first.

2.1 Compiling molBLOCKS in Linux and Mac OS X

In order to compile and install openbabel, make sure your system is capable of building C/C++ programs. Compilers can be readily obtained for both Mac OS X (Xcode development environment) and Linux. Open Babel also requires CMake, available for download at
http://www.cmake.org/cmake/resources/software.html

Then, please type the following:

```
# tar xzvf openbabel-2.3.2.tar.gz
# cd openbabel-2.3.2
# mkdir build
# cd build
# cmake ../
# make -j2
# sudo make install
```

For simplicity, we assume that the user has root privileges and can run the sudo command. Alternatively, a local installation is also possible.

Mac OS X users: to get OpenBabel to compile, you might find it helpful to get homebrew (http://brew.sh). Just type

```
# ruby -e "$(curl -fsSL https://raw.github.com/Homebrew/homebrew/go/install)"
```

at a terminal prompt.

Then this program can be used to obtain missing software. You may need to type the following at terminal prompts to get cmake and pkg_config, if you do not have them already:

```
# brew install cmake
# brew install Pkg_Config
```

As an alternative, MacPorts (http://macports.org/) can also be used to install cmake by typing:

```
# sudo port install cmake
```

cmake will be in the /opt/local/bin" directory.

For several Linux distributions it is also possible to install openbabel using the built-in packaging system, e.g. apt under Ubuntu Linux. Both the libopenbabel and libopenbabel-dev packages need to be installed in this case.

The next and final step is the compilation of the molBLOCKS suite. This is simply accomplished by entering the molblocks directory and typing make. In case of errors, it might be necessary to edit the path to the openbabel library in the Makefile by modifying the following line:

```
INCLUDES := -lboost -I/usr/local/include/openbabel-2.0
```

with the correct location of openbabel on your system.

2.2 Running molBLOCKS in a Virtual Machine

For users who do not wish to or cannot compile molBLOCKS, we prepared an image of Linux Debian with a pre-installed copy of molBLOCKS (http://compbio.princeton.edu/molblocks/download.html). Right-click (or control-click on Mac OS X) and download the .ova file containing the virtual machine image. The image should run out of the box on any virtualization environment, but we recommend VirtualBox (https://www.virtualbox.org/wiki/Downloads), which is freely available for Windows, Linux and Mac OS X.

After installing VirtualBox, double-click on the Linux image and import the Virtual Machine with standard settings. Alternatively, choose File→Import Appliance from Virtual Box menu. More information on importing a Virtual Machine can be found at https://www.virtualbox.org/manual/ch01.html#ovf.

After successfully importing the Virtual Machine, start it by pushing the play button. Once booted, the molBLOCKS program will be in the molblocks directory, ready for use. A README file in the login directory provides information on how to run the examples.

Using the fragment program
Input files
Parameters
Output
Under the hood

3 — The fragment program

The fragment program is used to break small molecules into chemically meaningful fragments. It requires a set of rules that define the bonds to be broken, and an input set of small molecules to fragment.

3.1 Using the fragment program

3.1.1 Input files

fragment requires two input files:

1. small molecules file in SMILES format (named molecules.txt here for convenience)
2. rules file defining the bonds to break in SMARTS format (e.g., RECAP.txt here)

An example of a molecules.txt file is the following:

```
Clc1ccc(C(N2CCN(CC2)CCOCC(=O)O)c2ccccc2)cc1 cetirizine
CC(=O)CC(C1=CC=CC=C1)C2=C(OC3=CC=CC=C3C2=O)O warfarin
...
```

Each line of the molecules.txt file contains exactly one molecule, entered as a SMILES string, followed by an optional name.

An example of a rule file (taken from the default RECAP rules that ship with molBLOCKS) is the following:

```
(\$([C!\$(C([\#7])(=O)[!\#1!\#6])](=[O]))]!@[\#7!\$([\#7][!\#1!\#6])]) amide
(\$(C=!@O))!@[\$([O;+0])]) ester
(\#6)!@(N;!\$(N=*);!\$(N(#6)=(!#6));!\$(N~(!#1!#6))!X4) amine
...
```

Each line in the rule file contains a description of a cleavable bond, in the form of a SMARTS pattern followed by an optional name. It is imperative that the SMARTS pattern define exactly the two atoms

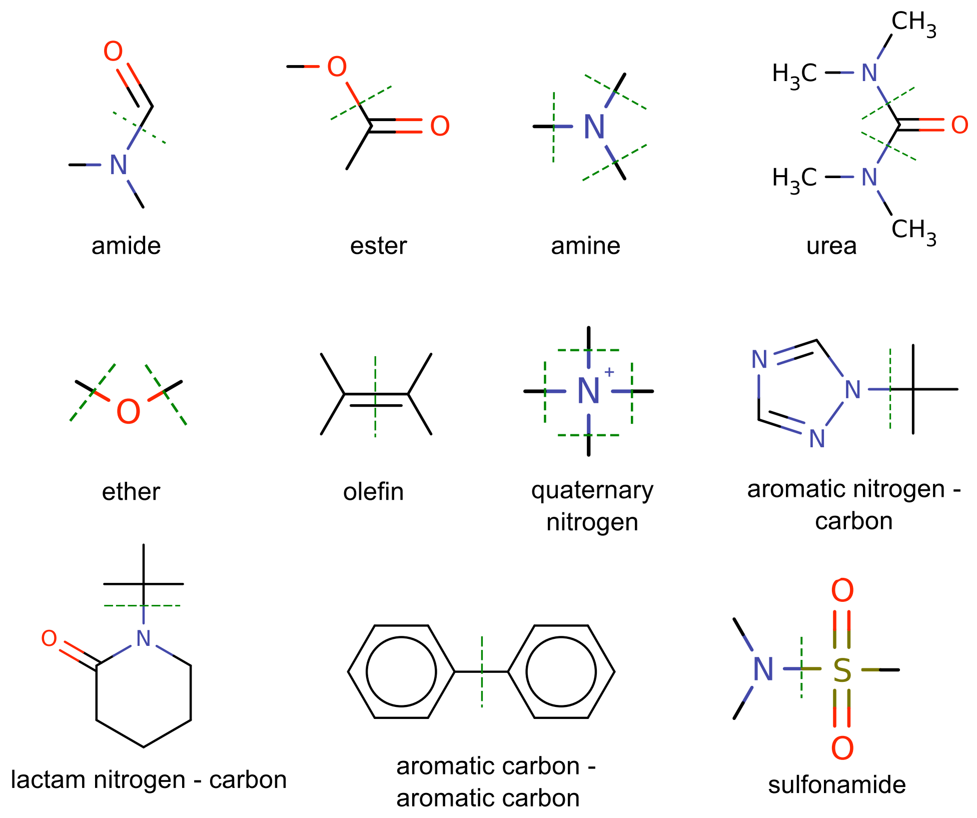

Figure 3.1: **RECAP rules.** The 11 rules that come by default with `fragment` were originally defined in [Lew+98], and specify all the bonds that can be cleaved by the `fragment` program. A user-defined set of rules can be used in place of the RECAP rules.

that form the chemical bond. Figure 3.1 shows the 11 rules that are used by default by `fragment`, obtained from the RECAP method [Lew+98].

`molBLOCKS` also provides the BRICS fragmentation rules[Deg+08] (file `BRICS.txt`), and the simple CCQ fragmentation rule implemented in the `MolFragment` program by ChemAxon, which cleaves a bond between two carbon atoms of which at least one is connected to a heteroatom (file `CCQ.txt`).

3.1.2 Parameters

The `fragment` program accepts the following parameters (the `optional` columns specifies whether a parameter can be omitted):

parameter	optional	example of argument	description
-i	N	input.txt	input file, one molecule per line
-o	N	output.txt	output file
-r	N	rules.txt	rules file, one rule per line
-n	N	4	minimum number of atoms in a fragment
-e	Y		flag to turn on extensive fragmentation

The `-e` flag turns on extensive fragmentation, and its use is recommended in order to get all the possible fragments from a molecule (see "Under the Hood" section for more details). The `-n` parameter specifies the minimum number of atoms that can be found in a fragment.
An example of a typical run:

```
# fragment –i molecules.txt –r RECAP.txt –e –n 4 –o fragments.txt
```

If the `-r` parameter is left out, the program will alert the user and automatically use the default RECAP rules.

3.1.3 Output

A typical example of an output (see Figure 3.2):

```
N(C@H)(C=O)CS.NCC(=O)O.O=CCC(C@@H)(C(=O)O)N
...
```

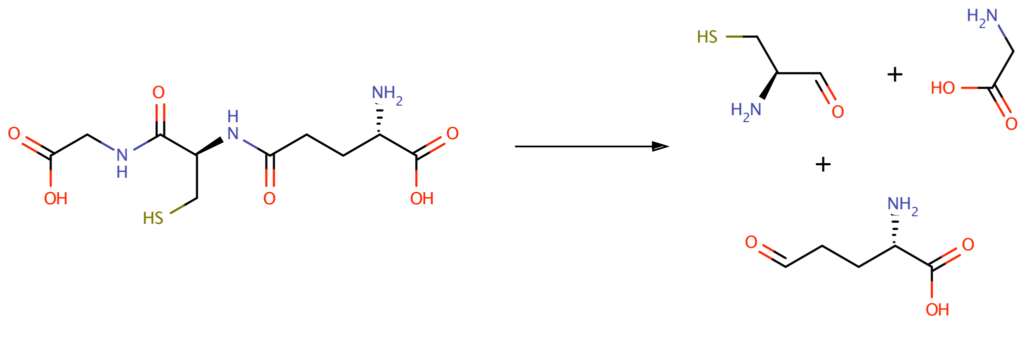

Figure 3.2: **Example of fragmentation.** The molecule to the left of the arrow is glutathione, broken by `fragment` into the three fragments on the right by applying the RECAP rules.

Each line in the output file will contain the fragments obtained by applying the fragmentation rules to the molecule, whose name (optionally) follows the fragments. Note that the fragments are separated by a period, the format used by SMILES to represent disconnected structures.

To visualize the output, the user can directly copy and paste the SMILES strings that contain the fragments into a visualization program, such as MarvinSketch (freely available at [1]), or a similar one. Figure 3.2 shows a visual representation of the fragments, obtained with MarvinSketch.

3.2 Under the hood

The main steps carried out by `fragment` with the extensive fragmentation flag (`-e`) turned on can be summarized as follows:

1. read the small molecules as SMILES strings
2. read the cleavage rules as SMARTS patterns
3. for each small molecule
   (a) identify all cleavable bonds in the molecule
   (b) build a graph representations of the cleavable bonds (see below), where there is an edge between cleavable bonds if they can be cleaved simultaneously
   (c) identify all the maximal cliques in the graph; these cliques can be overlapping
   (d) fragment the original molecule by breaking all the bonds in each maximal clique, one clique at a time

Handling of the SMILES and SMARTS strings is done through the Open Babel C++ API [OBo+11]. It is important to notice that not all bonds that match the rules can be cleaved at the same time, because doing so would yield fragments smaller than the minimum size. The *-e* flag ensures that all possible fragments are generated, using the following strategy. Cleavable bonds are represented as nodes in an undirected graph, with an edge between two nodes if both bonds can be cut (in other words, the bonds are independent from each other). Subsequently, the Bron-Kerbosch algorithm [BK73] is used to identify all maximal cliques. Finally, all the possible fragments are generated by cutting the bonds within each maximal clique, one clique at a time.

Without the `-e` flag, the bonds are applied sequentially, stopping as soon as no more fragments can be produced. In general, it is recommended to use the `-e` flag, unless dealing with particularly big molecules or if speed is at a premium. Fragmenting the entire DrugBank collection of 6460 small molecules took 53s (19s without the `-e` flag) on a iMac with a 2.66 GHz processor.

[1] http://www.chemaxon.com/products/marvin/marvinsketch/

Using `analyze`
Input files
Parameters
Output
A tutorial on fragment clustering and enrichment analysis
Under the hood
Fragment clustering
Enrichment analysis

4 — The `analyze` program

The `analyze` program is used to process the output of `fragment`, and it can generate statistics on fragment distributions, cluster the fragments by similarity, and perform enrichment analysis on a subset of small molecules.

4.1 Using `analyze`

4.1.1 Input files

`fragment` requires as input file the output of `fragment`, a simple text file containing one fragmented molecule per line. Optionally, a background file for enrichment analysis can also be supplied, in the same format as the input file. The input file should be a proper subset of the background file.

An example of an `input.txt` file is the following:

```
c1ccccc1.Nc1ncnc(n1)N 4429
Cn1ncc2c1ncnc2.NCc1ccccc1.Cn1ncc2c1ncnc2N.Cc1ccccc1 1451
...
```

4.1.2 Parameters

The `analyze` program accepts the following parameters (the `optional` columns specifies whether a parameter can be omitted):

parameter	optional	example of argument	description
-i	N	input.txt	input file, one set of fragments per line
-o	N	output.txt	output file
-c	Y	0.7	Tanimoto coefficient to cluster fragments
-e	Y	background.txt	background fragments for enrichment analysis

The `-c` option is used to perform fragment clustering, based on a Tanimoto similarity threshold between fragments.

The optional `-e` parameter specifies the background set that will be used for enrichment analysis. The background set **must contain** the main set of fragments (specified in the `input.txt` file). More information about fragment clustering and enrichment analysis can be found later in the tutorial and the "Under the hood" section.

An example of a typical run:

```
# analyze −i fragments.txt −e background.txt −c 0.7 −o distr.txt
```

If no argument is provided after the `-c` parameter, a default threshold of 0.8 will be used by the program.

4.1.3 Output

As an example, we show the five most frequent fragments in DrugBank, as reported by `analyze`:

```
261 c1ccccc1
149 Nc1ncnc2c1nc(nH)2
132 Cc1ccccc1
115 Oc1ccccc1
89 Nc1ccccc1
...
```

If all molecules in the input have an identifier, then the output will show all the molecules that contain the fragment:

```
261 c1ccccc1 DB00177 DB00251 DB00275 DB00349 DB00384 ...
```

In the example above, 261 molecules have a benzene ring, and their `DrugBank` IDs are shown in the last column. Please note that columns are always tab separated.
Clustering the fragments at a Tanimoto threshold of 0.7 yields the following top five representative fragments:

```
2837 CCCC(C@@H)(C(=O)O)N
1983 CC(Cc1ccc(cc1)O)N
411 Cc1c(nH)c2c1cccc2
```

```
274 Nc1ncnc2c1cccc2
261 Nc1ncnc2c1cccc2
```

As expected, the counts for the five most frequent fragments are now much higher, as the number of members in each cluster are summed up. The output with the `-e` enrichment option is:

```
p−value     FDR       Frequency   Fragment          Molecules
2.5e−03     5.1e−03   7           O=CCSc1ccncc1
6.4e−03     1.0e−02   3           C(C\#N)C=O
...
```

The first and second columns show the $p-$value and FDR, respectively. The third column shows the frequency of the given fragment (or its cluster), and the fourth column contains SMILES string of the fragment (or fragment representative). The last column shows which molecules have the fragment in question, provided that molecule identifiers are present in the input file.

4.2 A tutorial on fragment clustering and enrichment analysis

To illustrate how fragment clustering works, we briefly discuss the fragmentation and clustering of a set of nine cephalosporins, a widely prescribed class of beta-lactam antibiotics (Figure 4.1). The nine cephalosporins considered here are: cefacetril, cefaclor, cefadroxil, cefalexin, cefaloglycin, cefalotin, cefapirin, cefazolin, cefradin – see Figure 4.2 for their chemical structure. The input file containing the nine cephalosporins can be found in the `example` directory. The `test` directory contains the file that you should obtain after following this tutorial.

First, we need to fragment the cephalosporins and the background set, which contains all the small molecules (molecular weight <= 900 Daltons) in DrugBank [Wis+06]:

```
# ../fragment −i cephalosp.smi −r RECAP.txt −n 4 −o cephalosp.frag −e
# ../fragment −i background.smi −r RECAP.txt −n 4 −o background.frag −e
```

To cluster the fragments at a Tanimoto coefficient of 0.7 and compute enrichment analysis over the background distribution, type the following command:

```
# ../analyze −i cephalosp.frag −c 0.7 −o cephalosp.enrichCl07
              −e background.frag
```

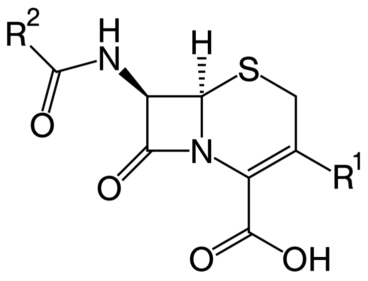

Figure 4.1: **Core structure of cephalosporins.** Substituent groups added at position R^1 and R^2 generate variants of the antibiotic.

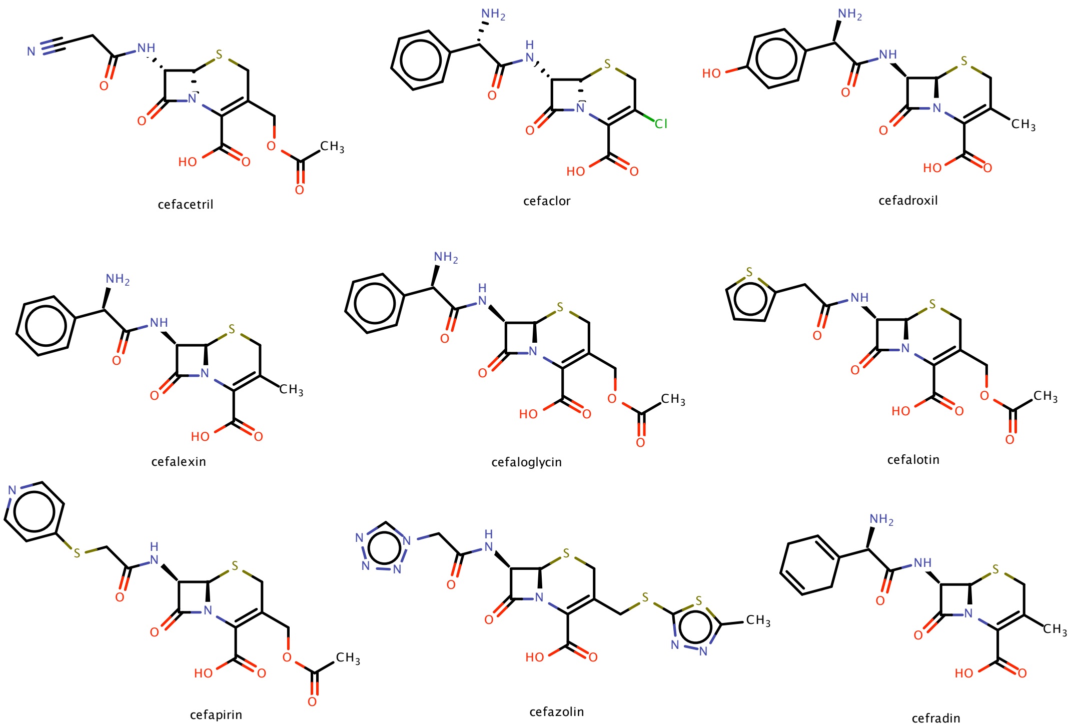

Figure 4.2: **Nine cephalosporins in DrugBank**

The resulting fragment distribution in shown in Figure 4.3. Note how the most frequent fragment representative closely matches the core structure of cephalosporins shown in Figure 4.1.

4.3 Under the hood

4.3.1 Fragment clustering

Fragments can be clustered using the `-c` option, followed by a Tanimoto coefficient that specifies the similarity threshold for clustering. In order to compute the similarity, fragments are converted to a fingerprint representation, based on linear segments of up to 7 atoms in length (FP2 fingerprints). The fingerprints are stored as bit arrays, where the presence or absence of a particular linear segment is represented by a 1 or 0, respectively. The FP2 fingerprint representation is obtained via the Open Babel library [1]. Then, the Tanimoto coefficient T_s between two fragments x and y is computed as:

Equation 4.1 $T_s = \frac{\sum_i X_i \wedge Y_i}{\sum_i X_i \vee Y_i}$ where X and Y are the bit array representations of the linear segments found in fragment x and y, respectively, and $\wedge$ and $\vee$ are the bitwise *and* and *or* operators.

The `analyze` program computes pairwise similarities between fragments and converts them to a graph representation, where an edge between fragments indicates a pairwise Tanimoto greater than the chosen threshold. Subsequently, the program extracts the connected components of the graph,

[1] http://openbabel.org/wiki/Tutorial:Fingerprints

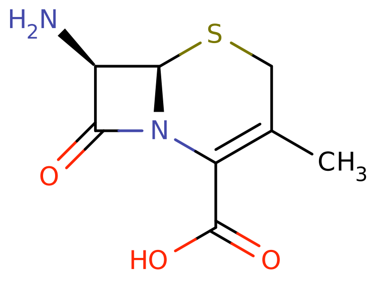

9/36

Figure 4.3: **Cephalosporin fragments clustered at a Tanimoto similarity of 0.7.** `analyze` returns one representative per cluster, by selecting the fragment with the highest average similarity to all the other fragments in the cluster. In the test case shown here, the only fragment occurring more than once with a significant FDR (FDR $< 2.2^{-16}$) is shown above. The representative fragment occurs nine times in the main set (once for each cephalosporin) and 36 times in the background (the entire DrugBank), and closely matches the core structure of cephalosporins shown in Figure 4.1.

and selects the representative element for each cluster as the fragment with the highest average similarity against all the other fragments in the cluster.

Clustering all the 15,532 fragments of DrugBank took 23s on an iMac with a 2.66 GHz processor.

4.3.2 Enrichment analysis

Enrichment analysis can be carried out in order to identify whether specific fragments (or clusters of fragments) appear in a set of molecules more frequently than expected by chance, given a background distribution. The hypergeometric distribution was chosen to model the probability of obtaining a number of fragments (or clusters of fragments) equal to or greater than the observed by chance alone (Equation 4.2).

Equation 4.2 $P(X \geq k) = \sum_{x=k}^{K} \frac{\binom{K}{x}\binom{N-K}{n-x}}{\binom{N}{n}}$

where N is the total number of fragments; K is number of fragments of the given type; n is the total number of fragments in the main set; and x is the total number of fragments of the given type in the main set.

`analyze` returns both uncorrected $p-$values and FDR corrected $p-$values, obtained with the procedure of Benjamini-Hochberg [BH95]. As mentioned before, the input set must be a subset of the background set for the results to be meaningful.

The results of enrichment analysis must be interpreted with caution, as a fragment that is present only once in the entire background set will show up as highly significant if present in the input set. Clustering the fragments reduces the number of unique or very rare fragments, but careful judgement is still needed.

Bibliography

[BH95] Yoav Benjamini and Yosef Hochberg. "Controlling the False Discovery Rate: A Practical and Powerful Approach to Multiple Testing". In: *Journal of the Royal Statistical Society. Series B (Methodological)* 57 (1995), pages 289–300 (cited on page 21).

[Ber+00] H. M. Berman et al. "The Protein Data Bank". In: *Nucleic Acids Res* 28.1 (2000), pages 235–42 (cited on page 6).

[BK73] Coen Bron and Joep Kerbosch. "Algorithm 457: finding all cliques of an undirected graph". In: *Commun. ACM* 16.9 (1973), pages 575–577 (cited on page 16).

[Deg+08] J. Degen et al. "On the art of compiling and using 'drug-like' chemical fragment spaces". In: *ChemMedChem* 3.10 (2008), pages 1503–7 (cited on page 14).

[Lew+98] X. Q. Lewell et al. "RECAP–retrosynthetic combinatorial analysis procedure: a powerful new technique for identifying privileged molecular fragments with useful applications in combinatorial chemistry". In: *J Chem Inf Comput Sci* 38.3 (1998) (cited on pages 5, 14).

[OBo+11] N. M. O'Boyle et al. "Open Babel: An open chemical toolbox". In: *J Cheminform* 3 (2011), page 33 (cited on pages 8, 11, 16).

[SLL02] Jeremy G. Siek, Lee-Quan Lee, and Andrew Lumsdaine. "The Boost Graph Library: User Guide and Reference Manual". In: (2002) (cited on page 11).

[Wei88] David Weininger. "SMILES, a chemical language and information system. 1. introduction to methodology and encoding rules". In: *J. Chem. Inf. Comput. Sci.* 28.1 (1988), pages 31–36 (cited on page 6).

[Wis+06] D. S. Wishart et al. "DrugBank: a comprehensive resource for in silico drug discovery and exploration". In: *Nucleic Acids Res* 34.Database issue (2006), pages D668–72 (cited on pages 6, 19).
