# Supplementary material for: molBLOCKS: decomposing small molecule sets and uncovering enriched fragments
Source: Bioinformatics. 2014 Mar 28;30(14):2081–3. doi: 10.1093/bioinformatics/btu173 (PMC4080744; doi:10.1093/bioinformatics/btu173)
Supplement: Supplementary Data [file supp_btu173_supplementary.pdf]

Supplementary Material

molBLOCKS: decomposing small molecule sets and uncovering enriched fragments

Dario Ghersi*, Mona Singh*†

1 Example of fragment enrichment analysis on a drug dataset

In order to present a proof-of-principle application of the molBLOCKS suite, we performed fragmentation and enrichment analysis on antineoplastic drugs, highlighting the statistically enriched fragments.

1.1 Dataset preparation

We collected all the drugs deposited in the KEGG DRUG Database[1, 2] (10,037 molecules). Of these, we selected the drugs that had at least one ATC (Anatomical Therapeutic Chemical Classification System) code, resulting in 4,581 molecules. Then, we filtered these compounds by molecular weight ($\leq$ 900 Daltons), obtaining the final background set of 3,685 small molecules. Antineoplastic drugs were extracted from the background set by selecting all the molecules annotated with the ATC code L01 ("antineoplastic agents"), resulting in 165 drugs.

1.2 Fragmentation

Both the background set and the antineoplastic set were fragmented using the `fragment` program with the RECAP rules[3], a minimum fragment size of 4 and the extensive fragmentation flag on. Extensive fragmentation ensures that all possible fragments are combinatorially generated whenever the bonds that match the rules cannot be cleaved all at the same time (please see the User's Guide for more details on the algorithm).

*Lewis-Sigler Institute For Integrative Genomics, Princeton University, Princeton, NJ 08544 USA
†Department of Computer Science, Princeton University, Princeton, NJ 08544 USA

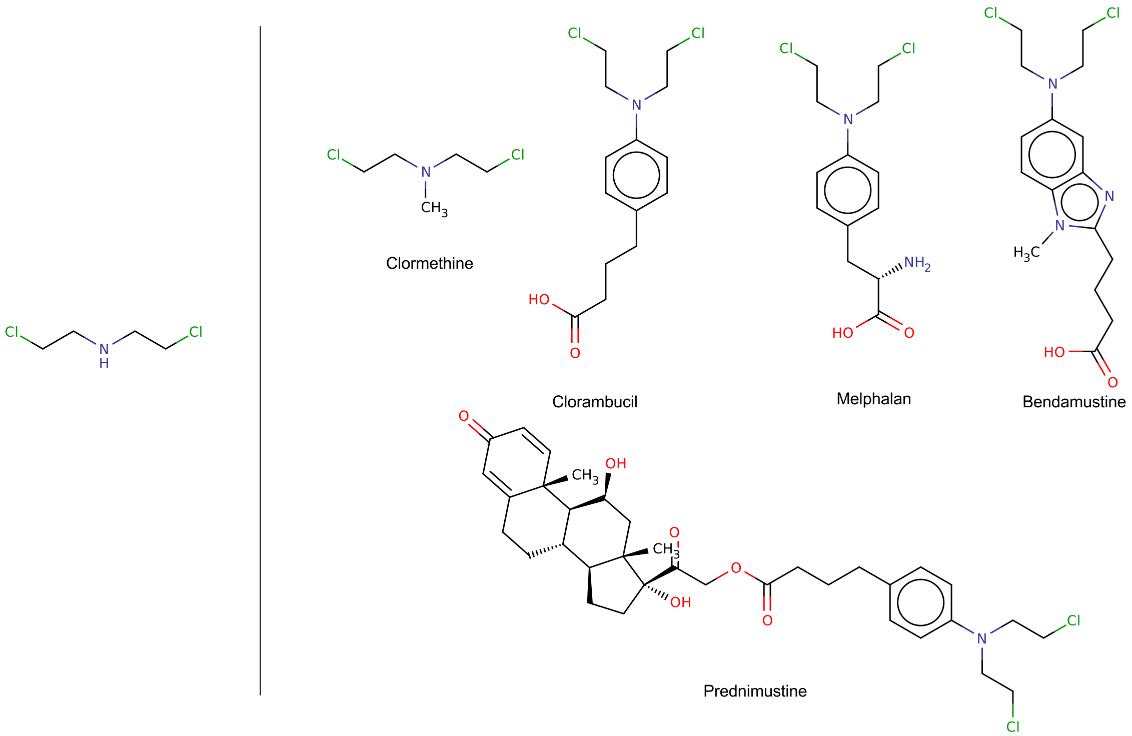

Figure 1: **Fragment 2** – The representative fragment for the second cluster is shown in the left panel, and drugs that contain a fragment in this cluster are shown in the right panel. The enriched fragment corresponds to chlormethine (minus the methyl group), the prototype drug of the nitrogen mustard alkylating agents, the class to which all the other compounds shown here belong.

1.3 Enrichment analysis

The `analyze` program was used to perform enrichment analysis on the antineoplastic set, clustering the fragments at a Tanimoto similarity of 0.8. Four clusters of fragments found to be significantly enriched (FDR $<= 0.001$) and together occurring > 5 times in the main set are highlighted in Figure 2 in the main text and Figures 1-3 here. We note that the `analyze` program found 2 additional enriched clusters that correspond to full molecules (i.e., they could not be fragmented according to the rules used), and 1 additional enriched cluster whose fragments overlapped those of one of the four main clusters (due to extensive fragementation). For convenience, these are not shown here.

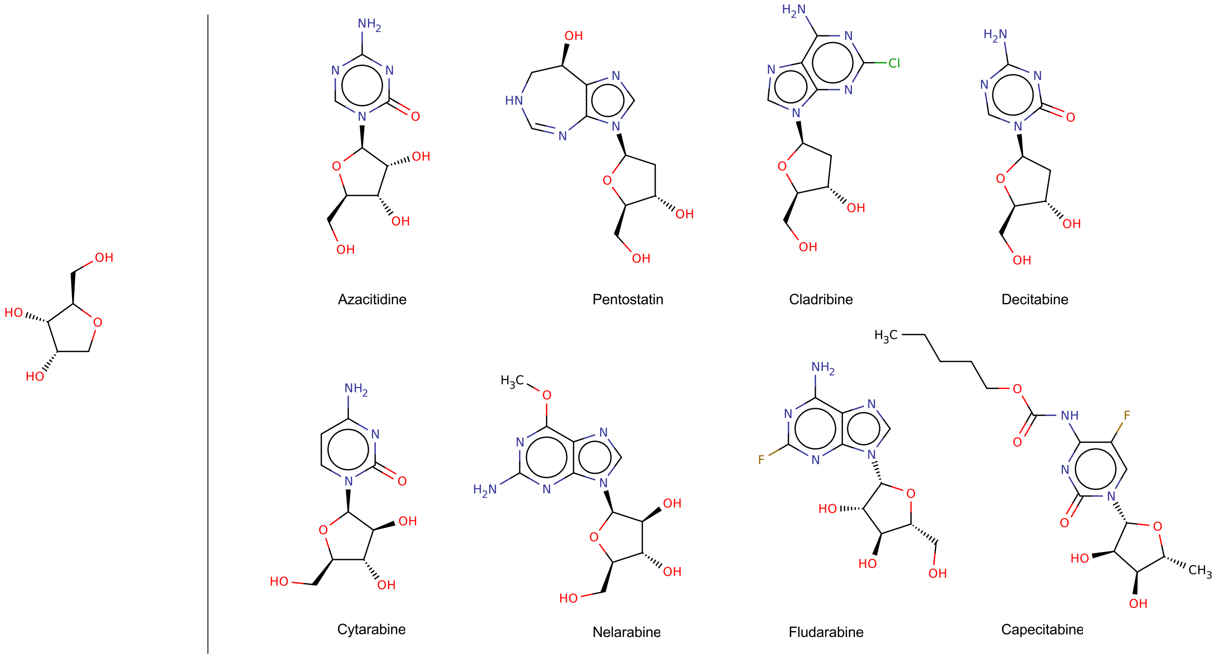

Figure 2: **Fragment 3** – All the compounds shown in the right panel are nucleosides, which act by mimicking DNA nucleobases, and interfere with DNA synthesis. The enriched fragment is the sugar moiety of the bases.

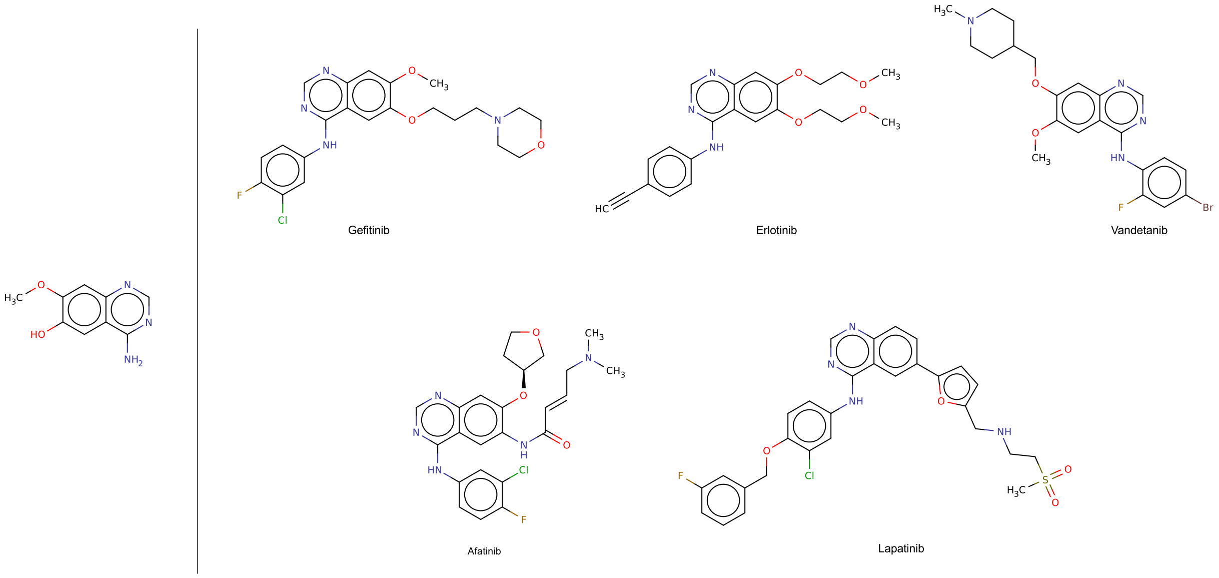

Figure 3: **Fragment 4** – The molecules shown here are all kinase inhibitors, and while different from each other at the global level, all share a heterocyclic aromatic nitrogen compound with a double-ring structure (quinoline). Quinoline has recently been called a "privileged" scaffold in cancer drug discovery [4].

References

[1] M. Kanehisa and S. Goto. Kegg: kyoto encyclopedia of genes and genomes. *Nucleic Acids Res*, 28(1):27–30, 2000.

[2] M. Kanehisa, S. Goto, Y. Sato, M. Furumichi, and M. Tanabe. Kegg for integration and interpretation of large-scale molecular data sets. *Nucleic Acids Res*, 40(Database issue):D109–14, 2012.

[3] X. Q. Lewell, D. B. Judd, S. P. Watson, and M. M. Hann. Recap–retrosynthetic combinatorial analysis procedure: a powerful new technique for identifying privileged molecular fragments with useful applications in combinatorial chemistry. *J Chem Inf Comput Sci*, 38(3), 1998.

[4] V. R. Solomon and H. Lee. Quinoline as a privileged scaffold in cancer drug discovery. *Curr Med Chem*, 18(10):1488–508, 2011.
